# Kiwi 4.0: In Vivo Real-Time Monitoring to Improve Water Use Efficiency in Yellow Flesh *Actinidia chinensis*

**DOI:** 10.3390/bios14050226

**Published:** 2024-05-03

**Authors:** Filippo Vurro, Luigi Manfrini, Alexandra Boini, Manuele Bettelli, Vito Buono, Stefano Caselli, Beniamino Gioli, Andrea Zappettini, Nadia Palermo, Michela Janni

**Affiliations:** 1 Istituto dei Materiali per L’Elettronica e il Magnetismo (IMEM-CNR), Parco Area delle Scienze, 37/A, 43124 Parma, Italy; filippo.vurro@imem.cnr.it (F.V.); manuele.bettelli@imem.cnr.it (M.B.); andrea.zappettini@imem.cnr.it (A.Z.); nadia.palermo@imem.cnr.it (N.P.); 2 Department of Agricultural and Food Sciences, University of Bologna, Viale Fanin 44, 40127 Bologna, Italy; luigi.manfrini@unibo.it (L.M.); alexandra.boini@unibo.it (A.B.); 3 Sysman Projects & Services Ltd., 70121 Bari, Italy; buono@sys-man.it; 4 CIDEA-UNIPR—Center for Energy and Environment, University of Parma, Parco Area delle Scienze, 95, 43124 Parma, Italy; stefano.caselli@unipr.it; 5 Institute of BioEconomy, National Research Council, 50145 Florence, Italy; beniamino.gioli@cnr.it

**Keywords:** plant monitoring, bioristor, OECT sensor, kiwifruit, water, precision irrigation, physiological responses

## Abstract

This manuscript reports the application of sensors for water use efficiency with a focus on the application of an in vivo OECT biosensor. In two distinct experimental trials, the in vivo sensor bioristor was applied in yellow kiwi plants to monitor, in real-time and continuously, the changes in the composition and concentration of the plant sap in an open field during plant growth and development. The bioristor response and physiological data, together with other fruit sensor monitoring data, were acquired and combined in both trials, giving a complete picture of the biosphere conditions. A high correlation was observed between the bioristor index (ΔI_gs_), the canopy cover expressed as the fraction of intercepted PAR (fi_PAR), and the soil water content (SWC). In addition, the bioristor was confirmed to be a good proxy for the occurrence of drought in kiwi plants; in fact, a period of drought stress was identified within the month of July. A novelty of the bioristor measurements was their ability to detect in advance the occurrence of defoliation, thereby reducing yield and quality losses. A plant-based irrigation protocol can be achieved and tailored based on real plant needs, increasing water use sustainability and preserving high-quality standards.

## 1. Introduction

Rising temperatures and the frequency of extreme weather events pose limits to agriculture production and severely impact food security. The reduced land availability and the recurrent drought events during the growing season hamper the quantity and quality of crop yields [1]. Due to the increased need for food security for an increasing world population [2], novel solutions in agriculture are needed to improve resource use efficiency and the resilience of crop systems to climate change as well as to optimize farm management and practices. In this scenario, the integration of advanced technologies based on novel sensors coupled with the Internet of Things (IoT) approach have the potential to positively impact agricultural production, minimize economic losses [3,4], and improve sustainability. 

Among horticulture crops, kiwifruit is grown in most temperate climates with adequate summer heat, representing an important market share [5]. Kiwifruit production in Italy is particularly important, being responsible for around 70% of the entire northern hemisphere production [6,7]. Italy is the second-largest kiwifruit producer in the world, with 562,188 metric tons cultivated annually (https://www.atlasbig.com/en-gb/countries-by-kiwi-production, accessed on 1 March 2024). A large percentage of its exports go to other European countries, including Germany, Spain, France, and Poland (https://www.atlasbig.com/en-gb/countries-by-kiwi-production, accessed on 1 March 2024). As with many fruit species, in kiwifruit, the plant water balance is a key factor that determines fruit growth and development, in particular, the balance between xylem and phloem inflows and/or water losses due to transpiration and xylem backflow [8]. In fact, Actinidia species originated in Southeast China under conditions of high precipitation and relative humidity [9]. Under these constraints, most of these species evolved large leaves (i.e., wide transpiring surface) and large and sparse xylem vessels, allowing a very high vine/stem hydraulic conductivity [10].

When the water requirement of leaves is sufficiently large to decrease the stem water potential to values lower than those of the fruit, some species like apple [11], kiwifruit [12], and grapevine [13] are subject to water loss due to xylem backflow. This phenomenon occurs mainly during the mid part of the season, when the xylem is still functional but is subsequently reduced, reaching zero close to harvest due to a loss in xylem functionality [14]. In kiwifruit, changes in the timing of irrigation can quickly affect the vine water relations, leaf gas exchange, and fruit vascular flow, finally impacting yield and production [15]. In this scenario, the development of smart plant monitoring solutions that are capable of detecting xylem flow and functionality can concretely improve resource use efficiency and can contribute to the development of novel decision support systems (DSS) [16] to fine tune irrigation practices.

Until now, remote sensing (satellite, airborne, or UAV platforms) and proximal sensing (multispectral or hyperspectral imaging, LiDAR, thermal imaging, or electromagnetic radiation) have been the most common used techniques concerning the acquisition of information about plant growth and health status [17,18]. However, being close to the plant but not directly embedded in it, both approaches give partial although reliable information on plant growth and development.

Recently, bioelectronic technologies offer new possibilities for real-time monitoring and dynamic modulation of plant physiology by translating complex biological inputs to electronic readout signals, while bioelectronic actuators can modulate biological networks via electronic addressing [19,20]. Portable plant items have started to emerge as epidermal sensors attached to the leaf or stem [21,22] and as more invasive devices implanted directly into the stem [20].

In 2017, a novel in vivo biosensor named bioristor was proposed [23]. Bioristor is an organic electrochemical transistor (OECT)-based biosensor that was originally applied to plants. The transistor is composed of a channel and a gate made of textile threads and functionalized with PEDOT/PSS [23]. The channel current can be modulated by the gate voltage that, when applied, pushes the positive ions of the electrolyte solution into the channel, changing its conductivity.

The relative variation in the channel current represents the sensor response (R), which is proportional to the concentration of ions dissolved in the plant sap [23]. Bioristor successfully monitored the changes occurring throughout the entire plant growth period, showing a high durability and accurate measurements both in vitro and in vivo [24,25]. It also demonstrated the ability to detect changes in the plant physiology mechanisms and the occurrence of water stress [25,26]. During a water deficit, a severe change in plant physiological mechanisms occurs, leading to response mechanisms such as reduced transpiration, stomatal closure, reduced photosynthesis, accumulation of ABA, and ion compartmentalization [26,27]. In a controlled environment, the in vivo monitoring by bioristor enabled the early detection of water stress [26]. By monitoring the changes occurring in ion concentrations in the plant sap flowing in the transpiration stream, bioristor was also successfully used for monitoring environmental changes such as VPD and relative humidity. In the field, bioristor was applied to monitor tomato crops throughout the entire production season, leading to the hypothesis that its adoption as a tool to guide irrigation could have led to 36% water savings [28]. Moreover, a high correlation between the bioristor R index and common vegetative indices used in field monitoring was observed, as well as a significant correlation with the crop stress water index [25]. Based on these results, bioristor was proposed as a tool for field phenotyping [25], coupled with a model based on artificial intelligence (AI) to forecast water stress in tomatoes [29]. Overall, bioristor proved to be able to monitor the functional physiology of apples, grapes, and kiwis [30].

Bioristor can concretely improve precision agriculture methods by being directly inserted in the plant, giving real time and continuous data on ion movement and compartmentalization and indirectly reporting on the plant water status continuously throughout the entire plant growth cycle. Based on these experiences, here, we assess the use of bioristor on gold kiwifruit to continuously monitor the plants during their growth and development under conventional irrigation management. This study is based on two years of monitoring in an open field within the framework of two research projects, POSITIVE and E-crops, both of which are focused on precision agriculture and encompass a range of environmental conditions (https://www.e-crops.it/, accessed on 1 March 2024, http://www.progettopositive.it/, accessed on 1 March 2024). The use of bioristor was originally combined with a physiological analysis and proximal soil sensors to analyze the water use efficiency. The novelty of this study resides in the assessment of the capability of bioristor to monitor the entire plant production cycle, identifying periods of drought stress and identifying novel correlations with other physiological indices. Moreover, the performance of the bioristor sensor in the presence of defoliation is assessed. 

## 2. Materials and Methods

### 2.1. Plants and Irrigation

A multi-site and multi-year approach was used to monitor kiwifruits using bioristor.

#### 2.1.1. First Year (2019)

The first trial was performed in Cesena (FC, Italy, coordinates 44.134225, 12.190178) within the framework of the POSITIVE project during the 2019 season. Two irrigation conditions were adopted, and sixteen bioristor were installed in two rows for each irrigation condition. Two sensors for each plant were installed on two two-years-old branches in different orientations by drilling a 0.75 cm hole using a microdrill. The installation took place during the fruit enlargement phase. 

Drip irrigation was utilized, and two irrigation conditions were defined: (i) farm irrigation (FI), corresponding to 100% water input and to the standard farm practice and (ii) modified irrigation (MI), corresponding to 120% water input (+20% with respect to the standard farm irrigation) using a microjet drip wing. 

#### 2.1.2. Second Year (2022)

The second experiment was conducted in 2022 in Southern Italy in Scanzano Jonico (MT, Italy, coordinates 40.337592, 16.720213) within the framework of the E-CROPs project. Three experimental plots (A, B, and C) were selected in a commercial orchard, and a bioristor sensor was mounted on one-years-old branches by drilling a 0.75 cm hole using a microdrill in two plots (B, C). 

A total of 4 sensors were installed in each plot on a total of 3 plants and connected to a control unit as previously described in Vurro et al., 2023 [30]. The sensors were powered by a 20 W solar panel and connected to two 12 V batteries to preserve autonomy. 

Irrigation was performed following the current scheduling (timing, volume) planned by the farmer to monitor the occurrence of possible water excesses and/or deficits during the growing season. A double irrigation system was set, integrating in-row localized drip lines (2.3 L/h) with a microjet sprinkler irrigation system (35 L/h) that was used alternatively by the farmer according to the specific weather conditions and water requirements.

### 2.2. OECT Sensor Device: Bioristor Preparation and Insertion in Trees

The bioristor sensors was fabricated, installed, and operated following previously reported methods [23,25,29]. 

**Figure 1:**
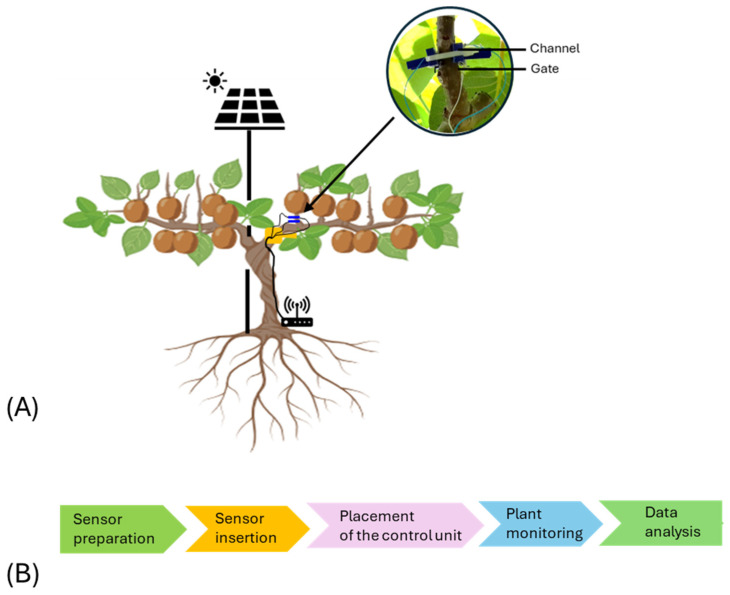
Bioristor system and implementation in kiwifruit. (**A**) Scheme of the bioristor sensor implemented in a kiwi plant, (**B**) Flowchart of the bioristor sensor monitoring in kiwifruit.

The OECT channel was inserted into the plant stem using an 0.8 mm drill and connected at both ends to a metal wire to form the source and the drain electrodes. Silver paste was used to secure the connections. The gate electrode, prepared using the same protocol, completed the design of the sensor device (Figure 1).

The bioristor signals were amplified using custom read-out electronics and connected to an IoT control unit based on the Arduino DUE system powered by a 12 V 12 Ah lead battery charged by a photovoltaic panel. The sampling rate was fixed at 1 Hz, and each control unit was able to read up to four sensors. The control unit was equipped with a 12-bit ADC (5 V full scale), the maximum current full scale was 7 mA, and the current resolution was about 1.5 μA.

A micro-weather unit was also incorporated into the control unit (DHT11 module, Seed Technology Inc., Shenzhen, China) to monitor the air temperature (°C) and relative humidity (RH).

A micro-SD memory card was used to collect the data in situ, and the data were also transferred to the cloud via a 4G connection. This allowed for the maximization of the signal-to-noise ratio using customized electronic circuits to amplify the bioristor signals, as well as for the local analysis of the raw data.

A constant voltage (V_ds_ = −0.1 V) across the transistor channel, along with a positive voltage at the gate (V_gs_ = 0.5 V), was applied to operate the bioristor sensor. The transistor current (I_ds_) and gate current (I_gs_) were monitored continuously throughout the entire duration of the experiments.

### 2.3. Measuring the Electric Activity of the Plants Using OECT

The operating principle of an OECT is thoroughly described in references [25]. The common nutrients absorbed through roots and circulating in the plant sap (NaCl, KCl, MgCl_2_, ZnCl, and other salts) dissociate as cations (M^+^) and anions (A^−^). In the OECT device, the channel is the active part, and it is made of PEDOT/PSS. Upon the application of a positive voltage at the gate, cations are forced towards the transistor channel, de-doping the PEDOT/PSS polymer. De-doping results in the removal of the charge carriers from the conducting polymer. The smaller number of holes available for conduction in the channel is a consequence of the incorporation of cations in the PEDOT:PSS. Cations entering into the PEDOT/PSS cause a reduction in the oxidized PEDOT^+^ and induce a decrease in the conductivity upon reduction to PEDOT. This de-doping process causes a reduction in the current from the drain to the source (I_ds_). The I_ds_ current is proportional to the cation concentration in the fluid.

The entire process is reversible. A voltage (V_ds_ = −0.1 V) was applied across the source and drain terminals of the channel, resulting in a continuous flow of current. In addition, a positive voltage was applied to the gate (V_gs_ = 0.5 V) for 15 min, causing a decrease in the conductivity of the channel due to the migration of cations from the electrolyte into the channel. When the gate voltage is switched off again for 15 min, the cations tend to return to solution through diffusion—this is the de-doping phase [31]. V_gs_ varies over time, following a typical 50% duty cycle square wave and periodically oscillating between 0 and 0.5 V with a frequency of two oscillations per hour.

In this configuration, the gate and the drain are the cathodes, and the source is the anode. Thus, positive charges move from the gate to the source and within the channel from the drain to the source.

The sensor response (R) is calculated as follows:

$$R=\frac{\left|I_{ds}-I_{ds0}\right|}{I_{ds0}}$$

which is proportional to the positively charged ion concentration.

I_ds0_ represents the current flowing across the channel when V_gs_ = 0.

At the same time, ΔI_gs_ that is the gate-source current variation, was also recorded in these trials as follows:

$${∆I}_{gs}=I_{gs}-I_{gs0}$$

which represents an overall estimation of the sap conductance.

I_gs0_ represents the current flowing across the channel when V_gs_ = 0.

ΔI_gs_ was found to be a key parameter in establishing the device saturation, i.e., the device wet fraction was correlated with the transpiration flux [28]. In some sense, while R is correlated with the sap ion concentration, ΔI_gs_ is related to the amount of sap that is wetting the device, reflecting the amount of sap that is circulating in the plant.

### 2.4. Physiological Measurements 

**Figure 2:**
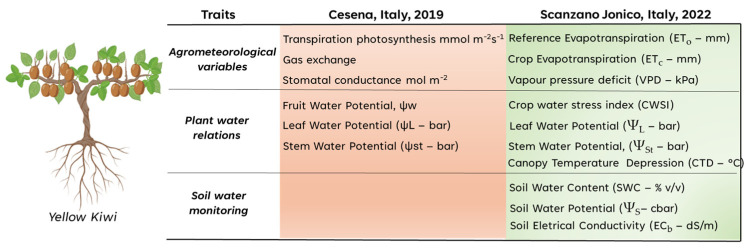
Physiological measurements performed in both locations and years (2019 and 2022).

Several measurements of key physiological traits were performed in both years and locations according to the instrument availability of the projects’ partners (Figure 2).

In 2019, physiological midday measurements were carried out within five monitoring periods of the fruit growing season between May and September: around 3, 8, 11, 15, and 20 weeks after full bloom (WAFB). The measurements consisted of plant water relations: stem water potential (Ψ_St_), leaf water potential (Ψ_L_), and fruit water potential (Ψ_F_); and plant gas exchanges: leaf photosynthesis (A_n_), stomatal conductance (g_s_), and transpiration. 

A Scholander pressure chamber (Soilmoisture Equipment Corp., Santa Barbara, CA, USA) was used to monitor the plants’ water status: Ψ_St_ was taken after isolating a leaf that was distant from the fruit and close to the trunk in ad hoc envelopes (aluminum foil on the outside and black on the inside) following the procedure described in reference [32]; Ψ_L_ was estimated from well-illuminated leaves that were distant from fruit; and Ψ_F_ was taken from representative fruits that were not fully exposed to light. 

A Li-COR 6400 (Li-COR 6400, LI-COR Inc., Lincoln, NE, USA) was used to estimate leaf gas exchange, including the stomatal conductance (g_s_), Leaf photosynthesis (A_n_) and transpiration were measured using a fluorimeter chamber that allowed for the measurement of the actual radiation reaching the orchard at the time of measurement.

In 2022, the complete set of agrometeorological variables (solar radiation, temperature, relative humidity, wind speed, rainfall) were measured along the cropping season, using the MeteoSense^TM^ Weather Station (Netsens, Ltd.). The data were integrated using the Bluleaf^TM^ DSS Software Platform (Bari, Italy) [33] to compute the components of the crop water balance according to the international standards established in the FAO Irrigation and Drainage Papers and more specifically the reference and crop evapotranspiration (ET_o_ and ET_c_, mm, respectively) and vapor pressure deficit (VPD, kPa) (Supplementary Figure S3). Moreover, the soil water status was monitored continuously in the three experimental plots (A, B, and C) using the following proximal sensors: (i) TerraSense^TM^ FDR soil moisture sensors (Netsens, Ltd., Calenzano, Firenze, Italy), measuring the dielectric constant and estimating the volumetric soil water content (SWC, % *v*/*v*); (ii) Watermark tensiometers, measuring the soil water potential (Ψ_S_, in the range of 0–200 cbars); (iii) ECSense^TM^ Sensors (Netsens, Ltd., Calenzano, Firenze, Italy), measuring the bulk electrical conductivity (EC_b_, dS/m). The soil sensors were positioned in the root zone at two depths, 0.25 and 0.50 m below the soil surface.

Additionally, a set of physiological parameters were evaluated in the experimental plots at 8, 10, 13, and 16 WAFB, with measurements taken around midday. First, the photosynthetic active radiation (PAR) was measured above and below the crop canopy on a regular grid of points designed on-row and between rows using a Quantum Light Meter (Spectrum Technologies Inc., Bridgend, UK). Then, the fraction of intercepted PAR (fi_PAR) was computed as the relative ratio between these measurements. 

The canopy temperature (CT, °C) was measured using a thermal infrared camera (FLIR TG-165X model) on both fully illuminated and shaded leaves. The measurements of CT were used to assess the following plant water stress indicators: (i) canopy temperature depression (CTD, °C), computed as the difference between air temperature (Ta, °C) and CT; (ii) crop water stress index (CWSI), following the original methodology proposed by Idso et al., 1982 [34] and setting the upper and lower baseline thresholds for kiwifruit according to an internal calibration performed during the E-CROPs project activities (data not shown). Finally, the Ψ_St_ and Ψ_L_ values were measured using a Scholander pressure chamber following the same procedure previously described for the 2019 trial. 

## 3. Results

### 3.1. Trial of 2019

**Figure 3:**
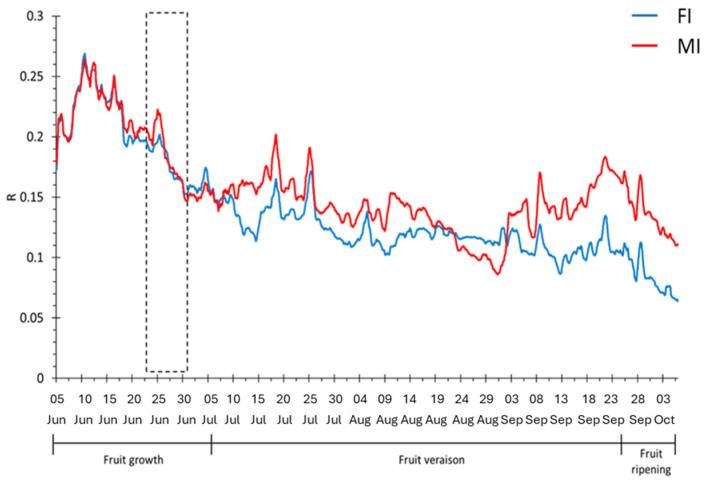
Sensor response in the 2019 trial. Dashed black block indicates the defoliation period. FI: farm irrigation, MI: modified irrigation.

The first yellow kiwi plant continuous monitoring was performed in Cesena for 4 months (Figure 3). The analysis of the bioristor sensor response (R) highlights a progressive R drop corresponding to a strong water deprivation, notwithstanding the high irrigation volumes provided. During the first month, no difference in the irrigation volume was observed, and the sensor response overlapped in values and slopes between the two water regimes for the entire initial period (5 June–6 July, in Figure 3).

After 6 July, the irrigation was differentiated, leading to a change in the R index trend that was consistent between the two irrigation regimes. 

**Figure 4:**
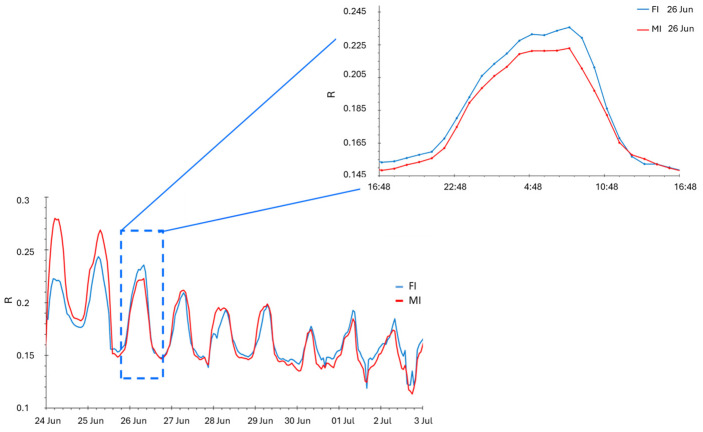
Plot of the daily sensor response during the defoliation period recorded on 26 June 2019. FI: farm irrigation, MI: modified irrigation.

Between 26 June and 30 June, a strong reduction in the sensor response was observed (Figure 3 and Figure 4), corresponding to a strong defoliation recorded in the same period.

**Figure 5:**
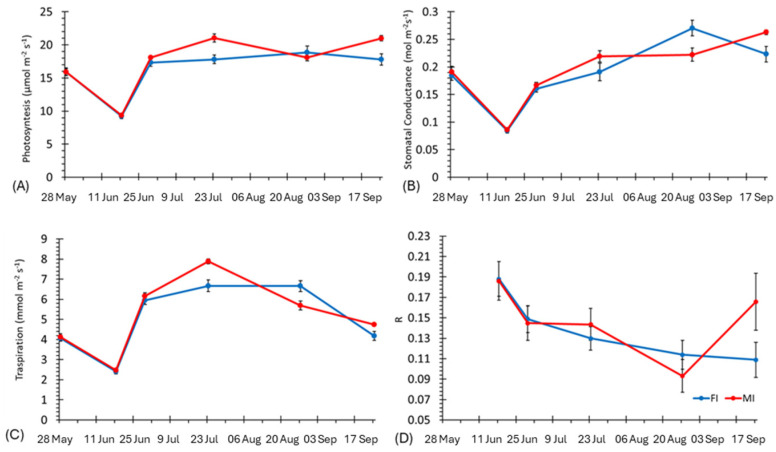
Plot of gas exchange traits and bioristor sensor responses acquired during the experiments of 2019. (**A**) Photosynthesis, (**B**) stomatal conductance, (**C**) transpiration, (**D**) sensor response. FI: farm irrigation, MI: modified irrigation.

A progressive drop in the sensor response was observed for both water regimes during fruit ripening, although it was more marked in the MI water regime. A similar behavior of the sensor response was previously observed in tomato plants during fruit maturation [28]. At a physiological level, an overall decrease in key mechanisms was observed in June, particularly for the stomatal conductance and transpiration of the plants in both water regimes (Figure 5B,C). From the end of July, a progressive increase in stomatal conductance and photosynthesis were observed, reaching higher values in MI (Figure 5). 

At the end of August, the maximum values of stomatal conductance were observed for FI, while photosynthesis remained stable in both water regimes. At the end of September, a decrease in the transpiration and steady values of photosynthesis and stomatal conductance were recorded.

**Figure 6:**
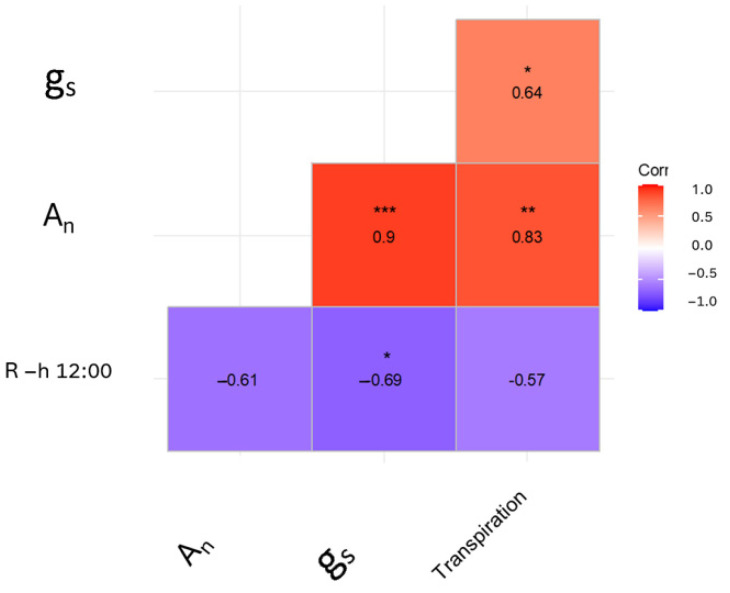
Correlation matrix of the sensor response and physiological parameters measured in the 2019 experiment. g_s_, stomatal conductance, A_n_ leaf photosynthesis, transpiration. * *p* ≤ 0.05, ** *p* ≤ 0.01, *** *p* ≤ 0.001.

A correlation analysis revealed a negative correlation of the sensor response observed at 12:00 a.m. with stomatal conductance (r = −0.69) and with transpiration (r = −0.57), contrary to what was observed in tomatoes, where a positive correlation between the conductance and sensor response was found (Figure 6, [26]).

### 3.2. Trial of 2022

**Figure 7:**
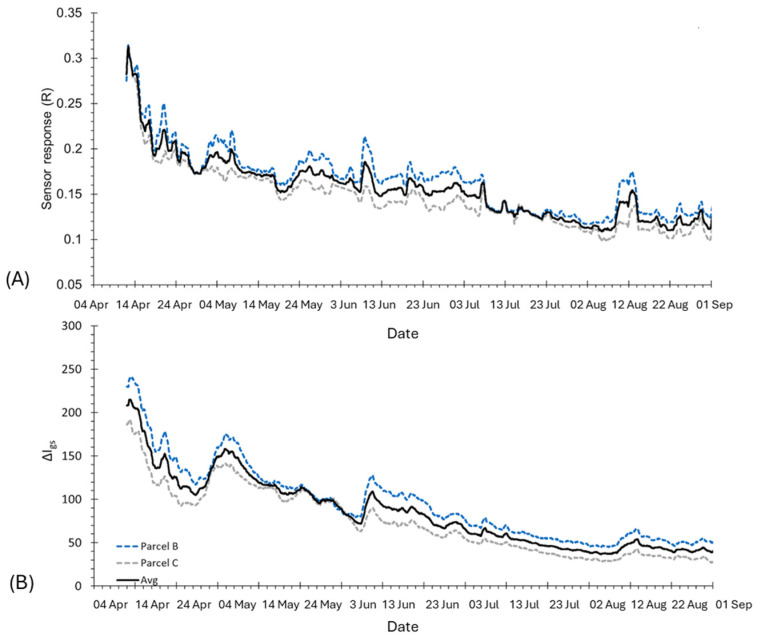
Plot of the bioristor indices. (**A**) Sensor response, R, (**B**) Gate-source current variation, ΔI_gs_ in time.

**Figure 8:**
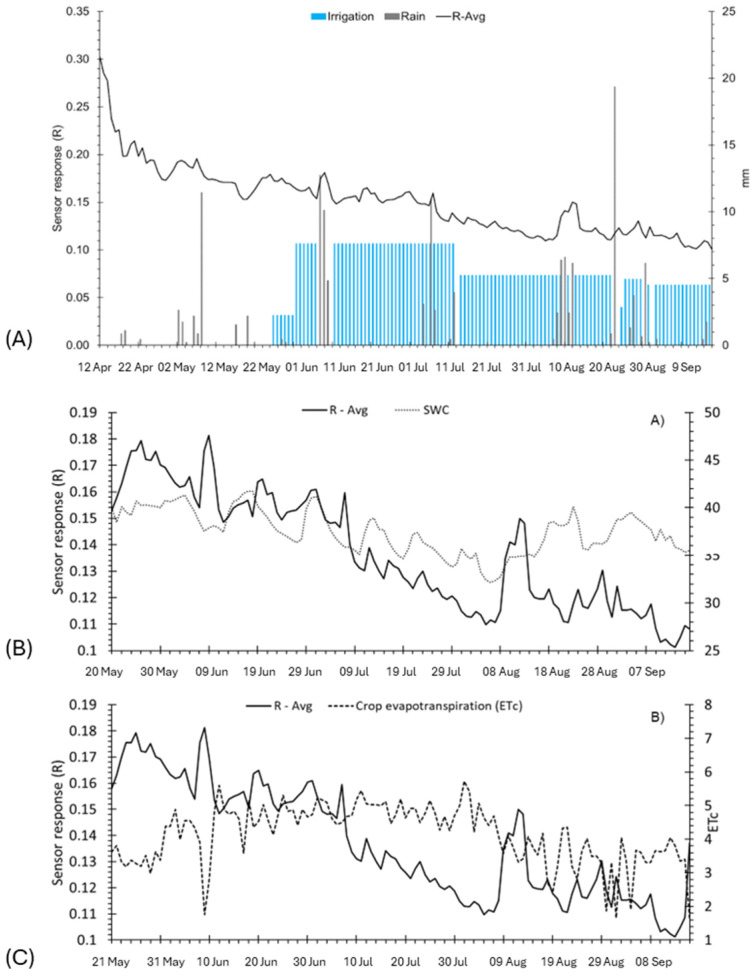
Plot of daily sensor response and key environmental traits: (**A**) rain (gray box) and irrigation (blue box), (**B**) soil water content (SWC), (**C**) crop evapotranspiration (ET_c_). R-Avg, R average; ET_c_ crop evapotranspiration.

To assess the efficacy of the bioristor sensors in kiwi plant monitoring, an additional trial was performed in Scanzano Jonico within the framework of the E-CROPs project. The plots of both R and ΔI_gs_ (Figure 7) showed a progressive, rapid, and strong drop of the signal during the initial phase of monitoring (April) that was related to the increase in crop evapotranspiration (Figure 8C) together with a substantial lack of rainfall and irrigation events (Figure 8A). In the first days of May, a partial recovery of the signal and several peaks were observed in relation to some rainfall events or irrigation, and similar peaks were also recorded later in June, July, and August. 

The irrigation season started at the end of May, with a constant daily water supply (of about 7.6 mm) from June to the middle of July that appeared to be sufficient to keep the R signal relatively stable while the ΔI_gs_ continued to decrease. After the middle of June, the daily irrigation supply was reduced to 5 mm, and this resulted in a corresponding decrease in both signals, reaching their minimum in the first week of August (Figure 8A).

**Figure 9:**
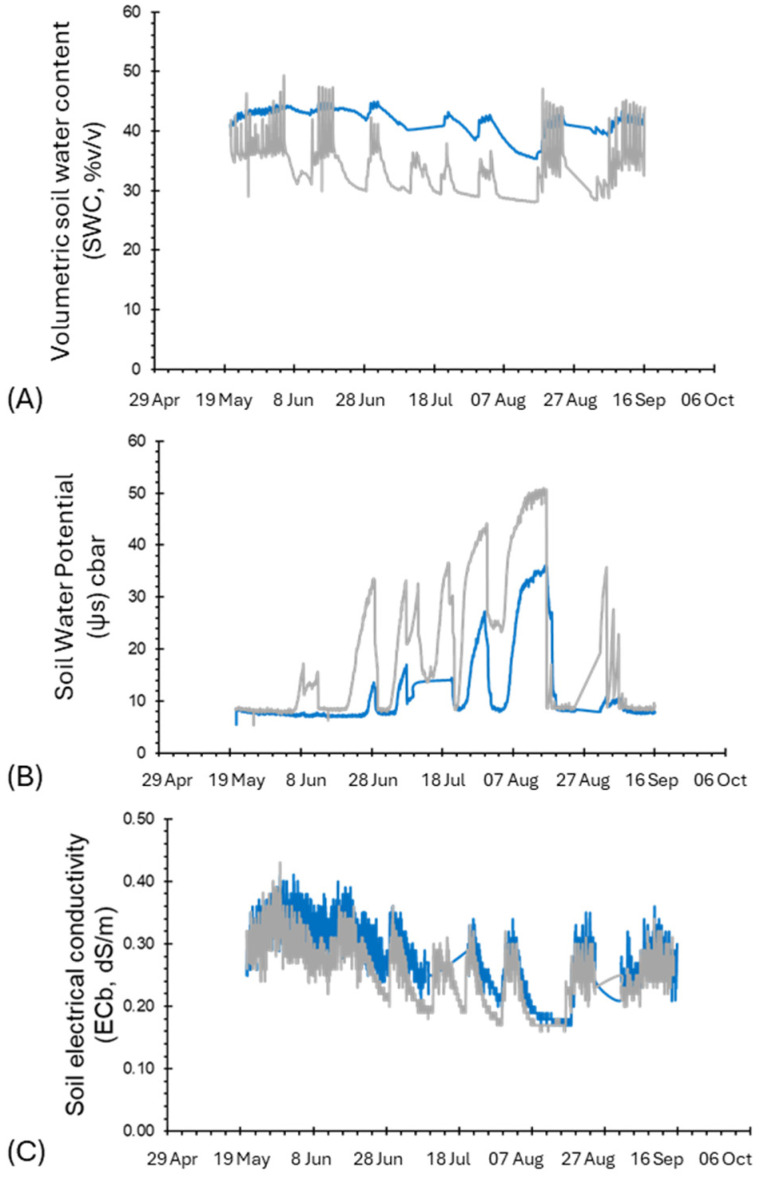
Plots of soil water content traits. (**A**) Volumetric soil water content (SWC, % *v*/*v*), (**B**) soil water potential (Ψ_S_, cbar), and (**C**) electrical conductivity, EC_b_. Blue lines indicate parcel B, and grey lines indicate parcel C. Data represent the average of the two depths of measurements for each parcel.

The time range between mid-May to September showed a similar trend in R and the soil sensors’ recorded humidity (Figure 8B and Figure 9A) until the first days of July, where the sensor response showed a strong decrease (Figure 8B,C). This indicated a redistribution of the ion content in the plant sap, reflecting the occurrence of stress conditions in the plant.

In relation to the data recorded by the soil sensors (Figure 9), the trends in the SWC and Ψ_S_ values were relatively stable from May to the middle of June. Then, a constant decrease in the SWC and a steady increase in Ψ_S_ were observed, with critical values reached from the end of July to the middle of August and a corresponding rapid and strong decrease in the R slope of both plots. Finally, a recovery pick of R signals was observed in both plots in correspondence with some rainy events occurring in the middle of August (10–14 August) and beginning of September (Figure 8A). Overall, a good agreement was also observed between the ECb measurements and the R signals, likely related to the corresponding variations in the concentration of ions in both soil and plants associated with the fluctuations in the soil water content (Figure 7A,B and Figure 9C).

**Figure 10:**
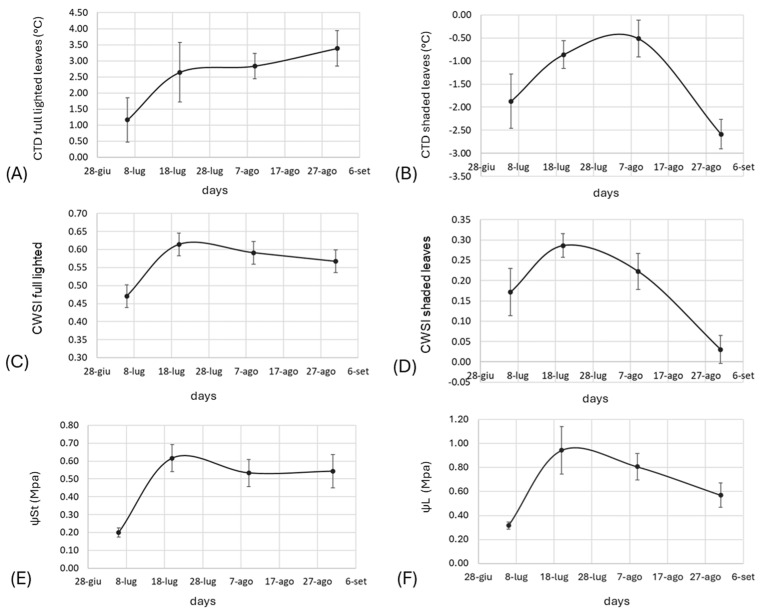
Plots of physiological traits recorded. (**A**) Canopy temperature depression, CTD, full-lighted leaves; (**B**) canopy temperature depression, CTD, shaded leaves; (**C**) crop water stress index, full lighted; (**D**) crop water stress index, shaded leaves; (**E**) stem water potential, Ψ_St_; (**F**) leaf water potential, Ψ_L_. Data represent the average of the tested plots, standard deviation is shown (n = 3).

In relation to the physiological measurements taken at 8, 10, 13, and 16 WAFB (Figure 10), increases across all the indicators of plant water stress were observed from the first to the second ten days of July, in agreement with the decrease in irrigation supply and soil water availability as well as the steady decrease in both bioristor indices (R and ΔI_gs_, Figure 7A,B).

More specifically, the CTD and CWSI indicators of full-lighted leaves and the Ψ_St_ values reached some critical threshold values that were recorded up to the end of August, as also supported by the R and ΔI_gs_ signal, suggesting the potential status of water stress of the plants due to an insufficient or inefficient irrigation water supply. At the same time, for the other indicators (CTD and CWSI of shaded leaves, together with Ѱ_L_), a partial recovery of the water stress was observed at the end of August, likely related to more favorable conditions in terms of soil water content and atmospheric water demand (ET_o_ and VPD) recorded in the last part of the season.

**Figure 11:**
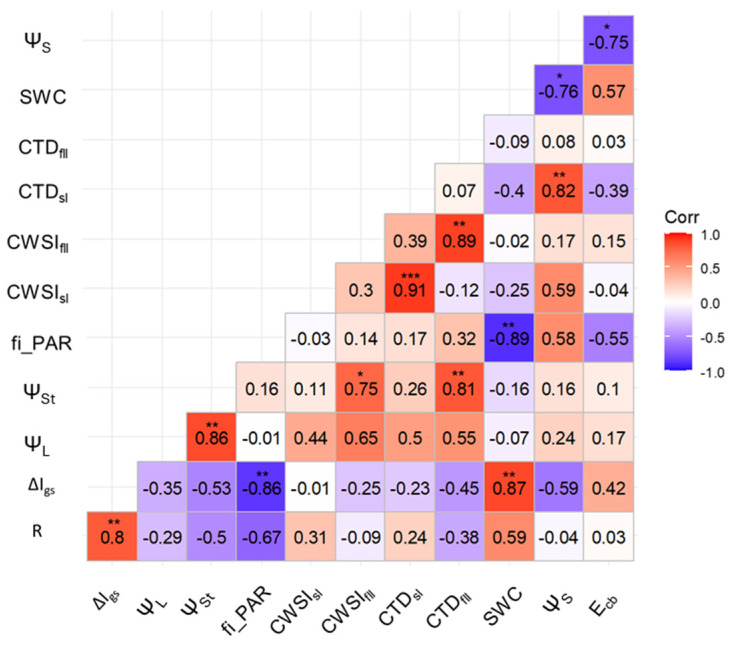
Correlation plot of all bioristor and physiological variables measured during the Scanzano Ionico trial on the days of 6 July, 20 July, 9 August, and 31 August. R, sensor response; ΔI_gs_ gate-source current variation; Ψ_L_, leaf water potential; Ψ_St_, steam water potential; fi_PAR, fraction of intercepted PAR; CTD_fll_, canopy temperature depression (full light leaves); CTD_sl_, canopy temperature depression (shaded leaves); SWC, soil water content; Ψ_s_, soil water potential; EC_b_, soil bulk electrical conductivity, CWSI_sl_, crop water stress index (shaded leaves); CWSI_fll_, crop water stress index (full light leaves). * *p ≤* 0.05, ** *p ≤* 0.01, *** *p ≤* 0.001.

The correlation matrix supported the draw hypothesis, showing a high correlation between ΔI_gs_, fi_PAR, and SWC, while R showed a good correlation with SWC, although this was not significant (Figure 11). CWSI in kiwifruit does not show a significant correlation with R as previously observed in tomatoes [25].

## 4. Discussion

In this study, two field trials are reported, exploiting bioristor deployment on yellow kiwifruits to perform functional phenotyping with a focus on water use and drought detection assessment given the high sensitivity of kiwifruit to water stress due to its low stomatal regulation. Bioristor sensors were used to detect periods of water stress in both seasons, with the final aim to drive optimized and sustainable irrigation, mitigate water stress, and increase intrinsic water use efficiency based on the early detection of stress conditions and the consequent definition of appropriate threshold values. 

The strength of bioristor is its ability to monitor the plant ions dynamic through the entire plant life cycle in vivo, in real-time, and continuously. We showed that the bioristor sensors enabled the detection of defoliation. This is supported by the analysis of the daily sensor response that was continuously reduced in intensity in both FI and MI (Figure 4) as a result of the reduced volume of the xylem sap [35] triggered by defoliation, together with a strong reduction in the whole-plant photosynthesis (Figure 5).

Defoliation can severely affect kiwi plants’ yields and health. These factors depend on (a) the intensity of defoliation; (b) type of tissue removed, and whether it is meristematic; (c) physiologic age; (d) frequency of defoliation, whether in discrete well-spaced events or continuous removal; (e) timing of defoliation; and (f) whether stresses or competition have occurred before, during, or after the defoliation [36]. Nevertheless, the higher percentage of defoliation observed in 2019 and the higher fruit damage due to sunburn in deficit-irrigated vines may indicate that the levels of water stress reached in this study were too severe for the kiwifruit. 

In previous studies, we have identified a strong link between the movements of water and ions in plant vessels and the bioristor index R [26]. Crop plants move water through the soil–plant–atmosphere continuum through the plant vascular system and lose it as latent heat through transpiration. Thus, the accuracy of in vivo monitoring of the ion movements in the transpiration stream is crucial to predict the use of water in these limiting environments.

In the kiwifruit, a strong anticorrelation between R and stomatal conductance was observed due to the stomata response to leaf water potential, which is one of the central elements of a plant’s drought response strategy [37,38,39]. This result differs from what was observed in tomato plants, where stomatal conductance and the R value were found to be positively correlated [26].

A strength of the bioristor measurements is the acquisition in real time of the sap ion concentration and its relationship with physiological acquired data, thus enabling the observation and prediction of water stress in cultivated plants. This observation is consistent with the findings of Vurro et al., 2023 [25], where R showed a specific and high correlation with the water-related indices (RWC, CWSI) that specifically traced the effects of drought stress.

A direct, real-time, and in vivo detection of the plant water status is highly significant for plant monitoring. In fact, it is well documented that many stomatal conductance-based models are biased because they overpredict stomatal conductance during conditions of low soil water potential, high VPD, and high leaf area [37]. The physiological data acquired in 2019 are in accordance with previously reported data that highlight that water potential (Ψ_St_) is strongly related to water stress [40,41] and decreases according to the level and duration of the drought treatment with corresponding decreases in soil humidity and a consequent increase in the temperature of the canopy [42].

Among the phenotypic traits, measuring the canopy temperature enables the detection of the water state of the plant and the balance between radical water absorption and transpiration [43,44]. Also, in this case, ΔI_gs_ is strongly negatively correlated with the canopy cover, expressed as fi_PAR and the soil water content (SWC) (Figure 10). The R value showed a moderate association with transpiration (r = −0.57), and a progressive drop in the sensor response (R) was observed in 2019 in both water regimes during fruit veraison. This was more marked in the MI (120%) water regime, in accordance with reference [45], which showed a strong reduction in the transpiration and xylem flux, and with reference [28], which showed a drop in the sensor response. In particular, after flowering, 90% of the water circulating in the xylem is lost due to transpiration due to the strong aptitude to lose water as a consequence of the numerous vital trichomes [45]. 

The combined analysis of the physiological stress indicators as CTD and CWSI of fully lit leaves, Ψ_St_, together with R and ΔI_gs_, indicated some critical threshold values that were recorded up to the end of August. This suggests a possibly critical water status due to insufficient or inefficient irrigation water supply [46]. However, in contrast with previously reported data on bioristor, no significant correlation was found between CWSI and R. This is likely due to the irrigation condition applied [28]. In tomatoes, a contrasting irrigation was applied between plots (100%, 80%, 40%), while in the Scanzano Jonico trial, a single irrigation condition was applied. 

The most commonly used index in guiding irrigation, SWC, showed a stable trend during fruit ripening, while the bioristor showed a progressive decrease in the sensor response, indicating the occurrence of a stress that could not be detected based on the SWC alone (Figure 7A, B). This finding is supported by previous work, reporting that limited SWC at the bud burst to flowering stage restricted vegetative growth [47], inhibiting the expansion and division of pulp cells under low SWC conditions at the fruit growth stage [48,49,50].

**Figure 12:**
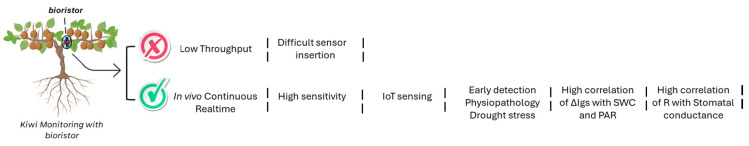
Advantages (√) and disadvantages (X)of the use of bioristor sensors in kiwifruit monitoring.

Thanks to the deployment of bioristor sensors, in kiwifruit, the continuous monitoring of plant development and the early detection of the onset of drought stress becomes feasible by tracking the sensor response R. Moreover, the observed strong correlation between SWC and ΔI_gs_ allows farmers to adjust the soil’s hydration status, addressing the needs of an optimized soil moisture level and supporting the importance of in vivo plant monitoring in fine tuning the irrigation recipe in kiwifruit (Figure 12).

## Data Availability

The data presented in this study are available upon request.

## Supplementary Materials

The following supporting information can be downloaded at: https://www.mdpi.com/article/10.3390/bios14050226/s1, Figure S1: Plot of the irrigations. Cesena, 2019; Figure S2: Plot of the sensor response and soil temperature monitoring. Cesena, 2019; Figure S3: Plots of environmental variables recorded in 2022.
