# Supplementary material for: Kiwi 4.0: In Vivo Real-Time Monitoring to Improve Water Use Efficiency in Yellow Flesh Actinidia chinensis
Source: Biosensors (Basel). 2024 May 3;14(5):226. doi: 10.3390/bios14050226 (PMC11117891; doi:10.3390/bios14050226)
Supplement: Supplementary file 1 [file biosensors-14-00226-s001.zip › biosensors-2942383-supplementary.pdf]

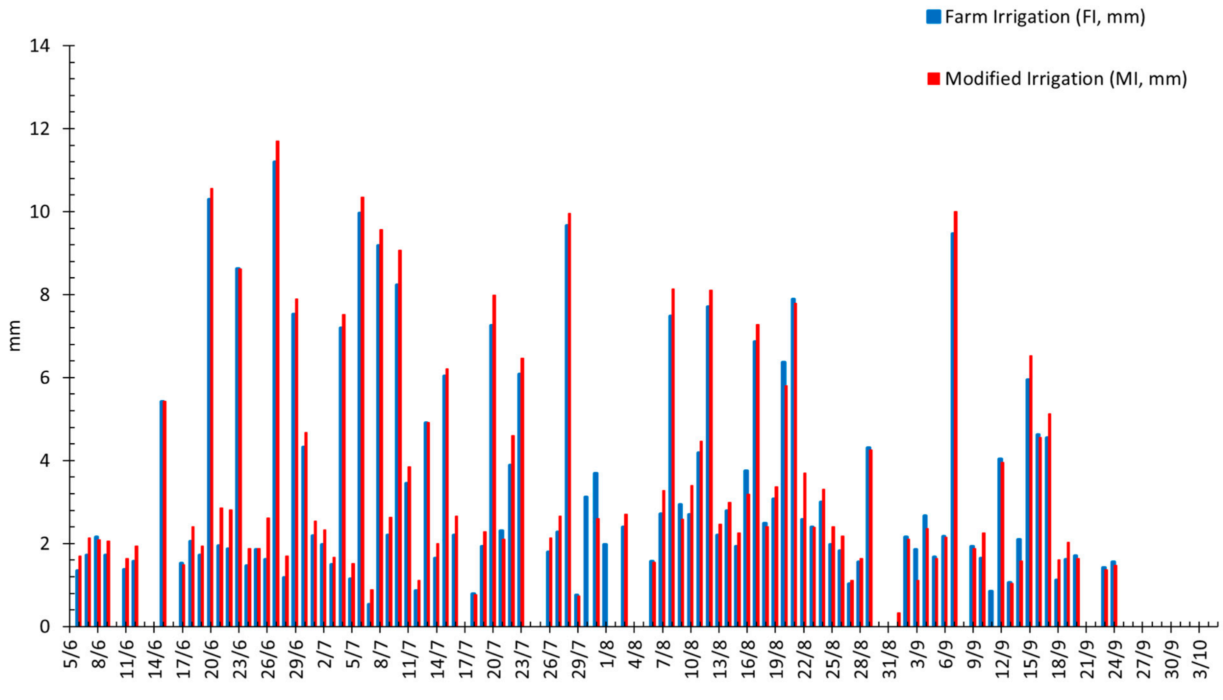

Figure S1. plot of the irrigations . Cesena, 2019.

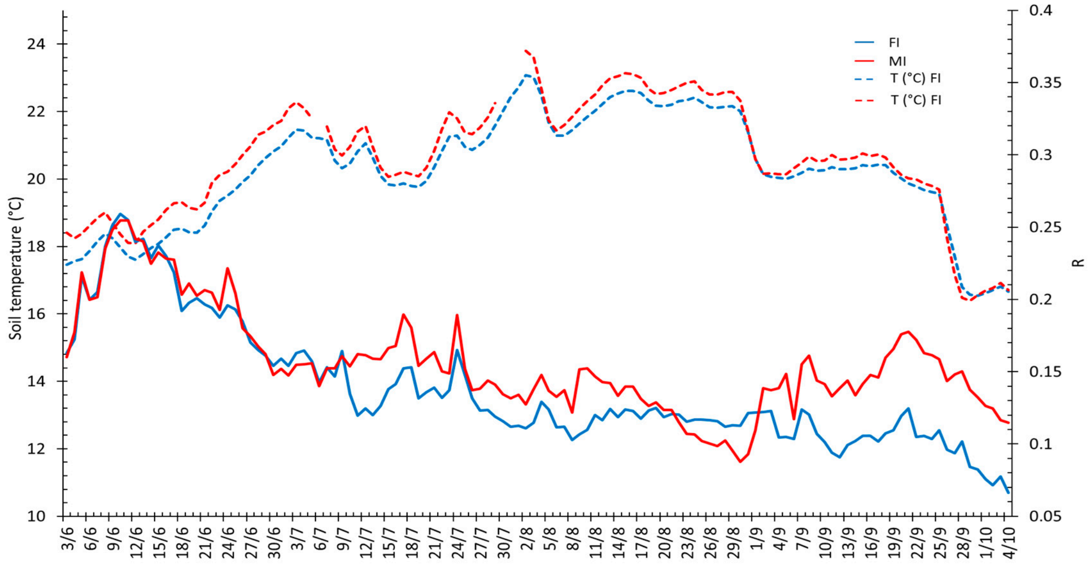

Figure S2. Plot of the sensor response and soil temperature monitoring. Cesena, 2019.

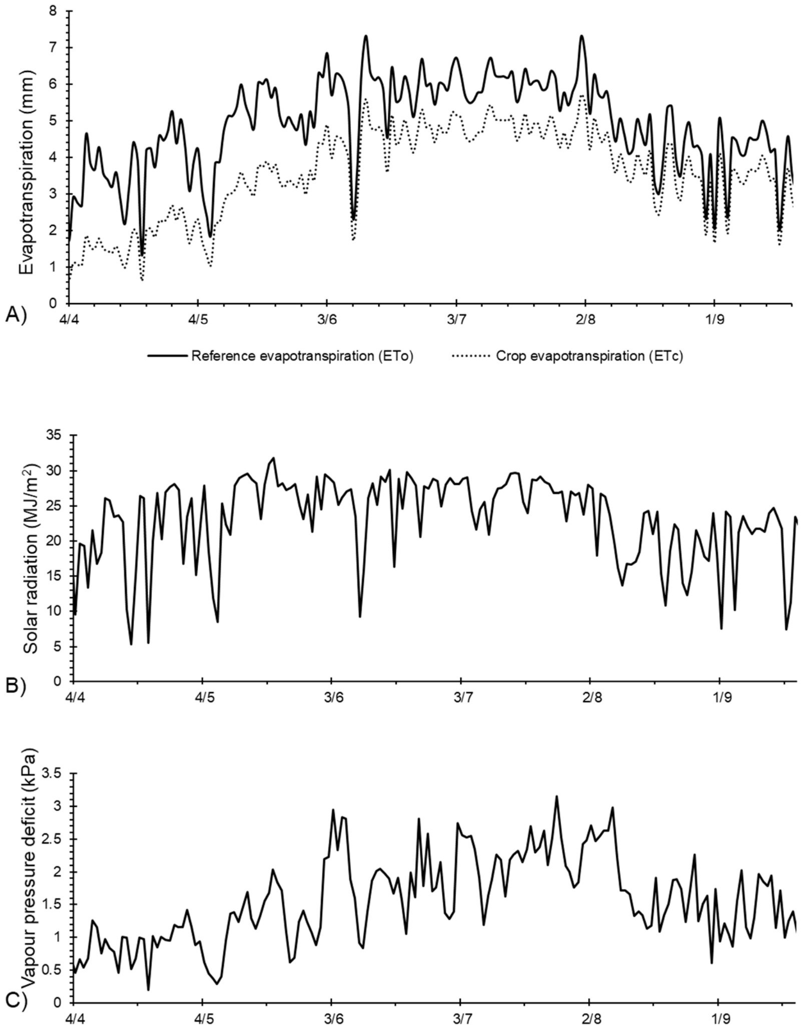

Figure S3. Plots of environmental variables recorded in 2022. A) crop evapotranspiration (mm), B) solar radiation (MJ/m^2), C) vapor pressure deficit (VPD, (kPa), as recorded in the experimental trial in Scanzano Jonico 2022 .
